# Family communication patterns and adolescent depressive symptoms: sequential mediating effects of hedonic capacity and anger expression

**DOI:** 10.3389/fpsyg.2026.1738349

**Published:** 2026-08-31

**Authors:** Feng Huang, Ning Hao, Wen-yi Xiao, Chi-son Kuan, Xin-li Chi, Chao Yan

**Affiliations:** 1 School of Psychology and Cognitive Sciences, Hefei Normal University, Hefei, China; 2 Key Laboratory of Philosophy and Social Science of Anhui Province on Adolescent Mental Health and Crisis Intelligence Intervention, Hefei Normal University, Hefei, China; 3 Shanghai Key Laboratory of Mental Health and Psychological Crisis Intervention, School of Psychology and Cognitive Science, East China Normal University, Shanghai, China; 4 Shanghai Changning Mental Health Centre, Shanghai, China; 5 School of Psychology, Shenzhen University, Shenzhen, China; 6 The Shenzhen Humanities & Social Sciences Key Research Bases of the Center for Mental Health, Shenzhen University, Shenzhen, China; 7 Key Laboratory of Brain Functional Genomics (MOE&STCSM), Affiliated Mental Health Center (ECNU), School of Psychology and Cognitive Science, East China Normal University, Shanghai, China

**Keywords:** anger expression, anhedonia, conformity orientation, conversation orientation, depression

## Abstract

**Objective:**

Depression is increasingly prevalent among adolescents, highlighting the need to explore its underlying developmental mechanisms to inform effective prevention. This study examined how different family communication patterns longitudinally impact adolescent depressive symptoms, with a focus on the mediating roles of diminished hedonic capacity and anger expression.

**Method:**

A total of 729 Chinese adolescents from five high schools participated in this two-wave longitudinal study over a six-month period. Participants completed the Family Communication Patterns Scale (FCPS), the Children’s Depression Inventory (CDI), the Anhedonia Scale for Adolescents (ASA), and the Children’s Inventory of Anger (ChIA). Mediation analyses were conducted using Hayes’ PROCESS macro, alongside cross-lagged panel analyses to explore longitudinal effect.

**Results:**

Cross-sectional analyses indicated that diminished hedonic capacity and anger experience sequentially mediated the relationship between family communication patterns and adolescent depressive symptoms. Specifically, this mediating effect existed for conversation orientation in junior high school students and female senior high school students, as well as for conformity orientation among female junior and senior high school students. In addition, longitudinal analyses revealed that diminished hedonic capacity mediated the associations at Wave 2.

**Conclusion:**

Our findings suggest that enhanced hedonic capacity and reduced anger expression may play a key role in the relationship between family communication patterns and adolescent depression, and hedonic capacity should be prioritized in practical interventions. These results support the potential value of interventions that promote positive family communication patterns as a means of mitigating adolescent depression, particularly for families with daughters.

## 1 Introduction

Depression is highly prevalent among adolescents, with a pooled prevalence rate of 23.2% for depressive symptoms (Racine et al., 2021), and 17.2% among early adolescents in China (Xu et al., 2020). Adolescent depression is linked to a range of adverse outcomes, including emotional disturbances, smartphone addiction, substance misuse, academic difficulties, sleep problems, and, in severe cases, suicidal ideation (Wu et al., 2024; Earnshaw et al., 2017; Yasugaki et al., 2025). Identifying both risk and protective factors is therefore crucial for informing effective prevention and early intervention strategies.

According to Family Communication Patterns Theory, two orientations—conversation and conformity—play a critical role in family functioning and can help predict a range of psychological outcomes (Koerner and Fitzpatrick, 2002). Conversation orientation refers to the extent to which families foster an open environment where all members are encouraged to engage in unrestricted dialogue. In contrast, conformity orientation reflects the degree to which families emphasize homogeneity in beliefs and attitudes. These orientations not only shape the emotional climate within families but are also closely associated with various aspects of mental health such as depression (Curran and Allen, 2016; Rueter and Koerner, 2008). However, few studies have systematically examined the mechanisms underlying the link between family communication patterns and adolescent depression (Curran and Allen, 2016; Curran et al., 2017). In particular, although depression is well known to be associated with diminished hedonic capacity and difficulties in emotion regulation—especially in managing anger (Xu et al., 2023; Everaert and Joormann, 2019; Sahu et al., 2014)—the specific roles these functions play in mediating the relationship between family communication patterns and depression remain unclear.

Anhedonia, defined as the reduced ability to experience joy from activities that are typically rewarding, is a core symptom of major depressive disorder (MDD) (Barkus, 2021). Studies have shown that approximately 70% of individuals with MDD experience anhedonia (Cao et al., 2019). Additionally, family communication plays a vital and far-reaching role in adolescent development. According to Family Communication Patterns Theory, distinct communication styles within families lead to different behavioral expectations (Koerner and Fitzpatrick, 2002). Adolescents raised in varying communication environments may differ in how they interpret others’ behaviors and, in their capacity, to learn through social interaction. Importantly, one key component of hedonic capacity is anticipatory pleasure—the ability to derive enjoyment from anticipating future rewarding experiences (Serretti, 2023). Drawing on these findings, it is reasonable to infer that adolescents from conversation-oriented families may develop more positive expectations due to open and supportive communication, thereby enhancing overall hedonic capacity (Powell and Hamilton, 2025). Conversely, a strong emphasis on conformity may limit emotional expression and reduce positive outcome expectations, ultimately diminishing adolescents’ hedonic capacity.

Additionally, anger expression, as a prominent form of negative affect, plays a critical role in the development and severity of depression (Judd et al., 2013). In understanding the relationship between family communication patterns and negative affect (e.g., anger expression), Bowen’s Family Systems Theory suggests that families function as emotional units, with members deeply interconnected through shared emotional dynamics (Carpenter et al., 2017). Adolescents raised in families with conversation-oriented patterns are more likely to experience a sense of security, predictability, and control in their daily lives, which in turn reduces the likelihood of negative emotional experiences (Carpenter et al., 2017). In contrast, a conformity-oriented family environment has been consistently linked to heightened expression of negative affect (Rueter and Koerner, 2008).

Furthermore, according to the Negative Affective Interference Theory (NAIT) and the Reward Devaluation Theory (RDT), individuals with depression often perceive positive external events as threatening, leading them to devalue future rewarding stimuli and ultimately reducing their hedonic capacity (Barkus, 2021). This diminished capacity, in turn, contributes to heightened negative affect, as previously rewarding experiences are reinterpreted as aversive. Therefore, it is plausible to infer a reciprocal relationship between hedonic capacity and anger expression. However, few studies have directly examined this link, particularly within the context of how family communication patterns contribute to adolescent depression, using both cross-sectional and longitudinal designs.

Sex differences in adolescent depression (Racine et al., 2021; Dong et al., 2025; Bowen et al., 2022) are well-documented. It is also found that the anhedonia level in female adolescents is significantly higher than in males (Watson et al., 2021). Regarding family communication patterns, research indicates that families with sons tend to exhibit more stereotypical conformity orientation compared to families with daughters (Koerner and Fitzpatrick, 2002). Additionally, some studies suggest that poor family relationships are more strongly associated with depression in adolescent girls than in boys (Chen and Yu, 2015; Zhou et al., 2023). Given these empirical findings, it is valuable to explore whether sex moderates the relationship among family communication patterns, hedonic capacity, anger expression, and depression.

A growing body of evidence indicates that the characteristics of depression, mental health, and suicidal ideation differ between junior and senior high school students (Liu et al., 2026; Wei and Liu, 2024; Zhang et al., 2024), for the reason that they differ in psychological maturity and developmental patterns (Chen et al., 2020). It was also found that parental democratic communication exerted a direct effect on junior high school student while it had only an indirect effect on senior high school students (Liu et al., 2024). Hence, it is necessary to examine the relationship between family communication patterns and depression separately for these two groups.

In this study, we aimed to investigate the longitudinal relationship between family communication patterns and depression among Chinese adolescents. Specifically, we sought to explore the mechanisms underlying this relationship through the perspectives of diminished hedonic capacity and anger expression, while also examining the moderating role of sex. All relationships were explored across two groups: junior and senior high school students. We hypothesized that conversation orientation would be negatively associated with adolescent depression, whereas conformity orientation would be positively associated with depression, and the relationship would be stronger among junior high school students. To further clarify the mechanisms underlying the relationship between family communication patterns and depression, we examined the sequential mediating roles of diminished hedonic capacity and anger expression. We hypothesized that diminished hedonic capacity and anger expression play a sequential mediating role in this relationship. Additionally, we anticipated that the effects of family communication patterns on depression, as well as the sequential mediating role of diminished hedonic capacity and anger expression, would persist over a 6-month period. Finally, we hypothesized that conformity orientation would be more strongly associated with depression in female participants than in male participants.

## 2 Materials and methods

### 2.1 Sample

Participants were recruited from five middle schools located in Baise (Guangxi Zhuang Autonomous Region), Chongqing, Zhaoqing (Guangdong Province), Huaibei (Anhui Province), and Chengdu (Sichuan Province). These five schools were selected via convenience sampling, and potential participants were invited through recruitment advertisements distributed by the schools. All participants completed online questionnaires under the guidance of trained research assistants. The baseline assessment included 1,004 adolescents aged 11 to 17 years (*M* = 14.00, SD = 1.68; 41.2% male), enrolled in grades 7 through 12. A total of 881 participants completed the second wave of data collection after 6 months. To control data quality, we included a check statement in the questionnaires that asks participants to choose the fixed selection (i.e., “select the ‘Sometimes’ option”). A total of 152 participants failed this statement, resulting in 729 valid participants (82.75%) remaining (*M* = 14.01, SD = 1.69; 38.1% male). Among them, 392 were junior high school students (*M* = 12.54, SD = 0.72; 41.8% male) and 337 were high school students (*M* = 15.75, SD = 0.63; 33.8% male). This study was approved by the Institutional Review Board (IRB: HR2-0066-2022) of East China Normal University before the recruitment process. Informed consent was obtained from both participants and their parents, who also consented to their child’s participation.

### 2.2 Instruments

#### 2.2.1 Family communication patterns scale (FCPS)

Family communication patterns were assessed using the Family Communication Patterns Scale (FCPS), originally developed by Ritchie and Fitzpatrick and validated in Chinese populations (Koerner and Fitzpatrick, 2002; Zhou et al., 2023). This 26-item measure comprises two subscales: Conversation Orientation (15 items) and Conformity Orientation (11 items). Higher scores on the Conversation Orientation subscale reflect greater openness and supportiveness in parent–child communication, whereas higher scores on the Conformity Orientation subscale indicate a stronger emphasis on uniformity and obedience within the family. Responses are rated on a 5-point Likert scale ranging from 1 (strongly disagree) to 5 (strongly agree). Cronbach’s alpha coefficients were 0.94 for conversation orientation and 0.89 for conformity orientation.

#### 2.2.2 Children’s depression inventory (CDI)

Depressive symptoms were measured using the Chinese version of the Children’s Depression Inventory (CDI), originally developed by Kovacs (2015) and adapted by Yu and Li (2000). The 27-item scale assesses depressive symptoms experienced over the past 2 weeks. Each item is rated on a 3-point Likert scale (1 = occasionally, 2 = often, 3 = always), with higher scores indicating greater depressive symptom severity. Cronbach’s alpha was 0.91 at both wave 1 (W1) and wave 2 (W2).

#### 2.2.3 Anhedonia scale for adolescents (ASA)

Diminished hedonic capacity was measured using the Chinese version of the Anhedonia Scale for Adolescents (ASA), originally developed by Watson et al. (2021) and adapted by Kuan et al. (2026). It is a 14-item scale comprising three dimensions: (1) enjoyment, excitement, and emotional flattening; (2) enthusiasm, connection, and purpose; and (3) effort, motivation, and drive. Participants responded on a 4-point Likert scale ranging from 1 (never) to 4 (always). Higher scores indicate lower hedonic capacity (i.e., more anhedonia). Cronbach’s alpha coefficients were 0.86 at W1 and 0.87 at W2.

#### 2.2.4 Children’s inventory of anger (ChIA)

Anger expression was assessed using the Chinese version of the Children’s Inventory of Anger (ChIA), originally developed by Nelson and Finch (2000) and Zhang et al. (2025). This 39-item measure includes four subscales: Frustration, Physical Aggression, Peer Relationships, and Authority Relations. Items are rated on a 4-point Likert scale (1 = do not care, 2 = bothers me, 3 = really mad, 4 = furious), with higher scores indicating greater anger expression. Cronbach’s alpha coefficients were 0.93 at W1 and 0.94 at W2.

### 2.3 Data analysis

We first tested the potential common method bias (CMB) that results from the use of self-report measures. We conducted a variance inflation factor (VIF) analysis by treating one of the predictor variables as the dependent variable and the remaining variables as independent variables in multiple regression models.

Then we analyzed the sociodemographic characteristics of the sample using SPSS 22.0. Pearson correlation analyses were then conducted to examine associations among age, sex, family communication patterns, diminished hedonic capacity, anger expression, and depressive symptoms at W1, as well as diminished hedonic capacity, anger expression, and depressive symptoms at W2. Next, we conducted cross-lagged panel analyses using structural equation modeling in Mplus 8.3, specifying bidirectional paths between diminished hedonic capacity and anger expression at both W1 and W2. Both cross-sectional and longitudinal mediation and moderation analyses were conducted using Hayes’ PROCESS macro version 3.3 for SPSS (Model 6 for sequential mediation and Model 92 for moderated mediation). In the cross-sectional analysis, family communication patterns were entered as independent variables separately, and depression symptoms at W1 were set as the dependent variable. Diminished hedonic capacity and anger expression at W1 were specified as mediators, while sex was included as the moderator in moderation analysis. In the longitudinal analysis, family communication patterns were entered as independent variables separately, and depressive symptoms at W2 were treated as the dependent variable. Diminished hedonic capacity and anger expression at W2 were specified as mediators, also in moderation analysis, sex was included as a moderator. Conversation orientation and conformity orientation were analyzed separately, given prior evidence of their inverse relationship (Keating, 2016) and their differential associations with adolescent depression (Curran and Allen, 2016; Curran et al., 2017). In the longitudinal analysis, we controlled for baseline depressive symptoms measured at W1. Both cross-sectional and longitudinal mediation and moderation analyses were conducted separately for junior high and senior high school students. Age was included as a covariate in all analysis. Bootstrapping procedures were used to estimate bias-corrected confidence intervals for indirect effects of family communication patterns on depressive symptoms at W2 via diminished hedonic capacity and anger expression.

## 3 Results

### 3.1 Common methods bias test

The results of the CMB test indicated that all VIFs ranged from 1.09 to 2.38, and all tolerance values exceeded 0.41, suggesting no multicollinearity or significant CMB. Further assessment using Harman’s single-factor test revealed that the single-factor model exhibited poor fit (*RMSEA* = 0.088, *CFI* = 0.477, *TLI* = 0.459, *SRMR* = 0.106). Similarly, the four-factor model—which grouped conversation and conformity orientations together—also showed unsatisfactory fit (*RMSEA* = 0.067, *CFI* = 0.699, *TLI* = 0.687, *SRMR* = 0.080). In contrast, the five-factor model, which treated conversation and conformity orientations as separate constructs, demonstrated an acceptable fit (*RMSEA* = 0.049, *CFI* = 0.843, *TLI* = 0.837, *SRMR* = 0.053). The findings indicate that CMB is not a substantial concern in this study.

### 3.2 Descriptive statistics and correlations

Table 1 presents the descriptive statistics and correlation coefficients for all study variables. The results indicated that both conversation-oriented and conformity-oriented family communication patterns were significantly correlated with ASA total scores, ChIA total scores, and CDI total scores at both at W1 and W2 (*ps*<0.01).

**Table 1:** Descriptive statistics and correlations (*n* = 729).

Variable	*M*	*SD*	sex	age	Conversation	Conformity	ASA W1	CDI W1	ChIA W1	ASA W2	CDI W2	ChIA W2
Sex	–	–	–									
Age	14.02	1.74	−0.04	–								
Conversation	3.29	0.80	0.07	−0.11^**^	–							
Conformity	2.79	0.72	0.07	0.03	−0.28^**^	–						
ASA W1	1.82	0.45	−0.07^*^	0.15^**^	−0.47^**^	0.24^**^	–					
CDI W1	1.53	0.33	−0.11^**^	0.19^**^	−0.46^**^	0.28^**^	0.73^**^	–				
ChIA W1	2.07	0.42	−0.13^**^	0.003	−0.25^**^	0.15^**^	0.24^**^	0.35^**^	–			
ASA W2	1.94	0.46	−0.10^**^	0.11^**^	−0.37^**^	0.20^**^	0.63^**^	0.57^**^	0.19^**^	–		
CDI W2	1.55	0.32	−0.16^**^	0.10^**^	−0.35^**^	0.23^**^	0.58^**^	0.68^**^	0.22^**^	0.79^**^	–	
ChIA W2	2.09	0.44	−0.13^**^	−0.02	−0.21^**^	0.09^*^	0.22^**^	0.25^**^	0.52^**^	0.27^**^	0.33^**^	–

***p < 0.001, **p < 0.01, *p < 0.05.

W1, baseline; W2, six-month follow up. Sex was coded as 0 for female and 1 for male in the correlation analysis.

ASA, the Anhedonia Scale for Adolescents; CDI, the Children’s Depression Inventory; ChIA, the Children’s Inventory of Anger.

### 3.3 The cross-lagged analysis of hedonic capacity and anger expression

To explore the sequential mediation role of ASA scores and ChIA total scores in the relationship between family communication patterns, we firstly examined reciprocal relationship between ASA and ChIA total scores. As shown in Table 1, sex was negatively associated with ASA and ChIA total scores at both W1 and W2, while age was positively associated with ASA total scores at both waves. Accordingly, we controlled for the effects of sex and age on ASA total scores, and for the effects of sex on ChIA total scores in the structural equation model.

The model demonstrated an acceptable fit to the data (*RMSEA* = 0.09; *CFI* = 0.89; *TLI* = 0.87; *χ^2^* = 5644.17, *χ^2^/df* = 47.43, *SRMR* = 0.06). ASA total scores at W1 significantly predicted ASA total scores (*β* = 0.68, *p* < 0.01) and ChIA total scores (*β* = 0.12, *p* < 0.01) at W2. However, the effect of ChIA total scores at W1 on ASA total scores at W2 was not significant (*β* = 0.01, *p* = 0 0.74). Additionally, significant correlations were observed between ASA and ChIA total scores at both W1 (*r* = 0.28, *p* < 0.01) and W2 (*r* = 0.22, *p* < 0.01).

### 3.4 The cross-sectional and longitudinal mediating role of RMSEA hedonic capacity and anger expression

Building on the structural equation model results, we conducted a sequential multiple mediation analysis using Hayes’ PROCESS Macro for SPSS (Model 6) to examine the mediating roles of diminished hedonic capacity and anger expression in the relationship between family communication patterns and depressive symptoms among junior and senior high school students separately, given the notable differences in adolescents’ physical and psychological characteristics across the two academic stages (Wei and Liu, 2024; Zhang et al., 2023). As shown in Table 2, for junior high school students, conversation orientation score of FCPS had a direct negative effect on CDI scores (point estimate = −0.0725, *95% CI* = −0.1047 to −0.0403). The ASA total scores (point estimate = −0.1132, *95% CI* = −0.1422 to −0.0873) and ChIA total scores (point estimate = −0.0101, *95% CI* = −0.0198 to −0.0020) each independently mediated the effect of conversation orientation scores on CDI scores. A significant sequential mediating effect emerged through ASA and ChIA total scores, indicating that greater conversation orientation predicted enhanced hedonic capacity, which in turn was associated with reduced anger expression and ultimately lower depressive symptoms (point estimate = −0.0075, *95% CI* = −0.0162 to −0.0020). For senior high school students, the ASA total scores (point estimate = −0.1202, *95% CI* = −0.1547 to −0.0875) and ChIA total scores (point estimate = −0.0141, *95% CI* = −0.0289 to −0.0030) separately mediated the effect of conversation orientation scores on CDI scores. However, the direct effect of FCPS conversation orientation scores on CDI scores (point estimate = −0.0213, *95% CI* = 0.1630 to −0.0513) and the sequential mediating effect via ASA and ChIA total scores (point estimate = −0.0033, *95% CI* = −0.0082 to 0.0019) were non-significant.

**Table 2:** Analysis of model mediating effect of family communication patterns based on cross-sectional data.

School stage	Family communication patterns	Path	Effect	Boot SE	Boot LLCI	Boot ULCI	Effect proportion
Junior high school students	Conversation orientation	Total effect	−0.2033^***^	0.0175	−0.2377	−0.1689	
		Direct effect	−0.0725^***^	0.0164	−0.1047	−0.0403	35.66%
		Indirect effect	−0.1308^***^	0.0156	−0.1619	−0.1002	64.34%
		Conversation orientation → ASA W1 → CDI W1	−0.1132^***^	0.0141	−0.1413	−0.0861	55.68%
		Conversation orientation → ChIA W1 → CDI W1	−0.0101^*^	0.0047	−0.0201	−0.0019	4.97%
		Conversation orientation → ASA W1 → ChiIA w1 → CDI W1	−0.0075^*^	0.0034	−0.0154	−0.0021	3.69%
	Conformity orientation	Total effect	0.1366^***^	0.0213	0.0948	0.1784	
		Direct effect	0.0676^***^	0.0159	0.0364	0.0988	49.49%
		Indirect effect	0.0690^***^	0.0163	0.0384	0.1023	50.51%
		Conformity orientation → ASA W1 → CDI W1	0.0549^*^	0.0145	0.0271	0.0840	40.19%
		Conformity orientation → ChIA W1 → CDI W1	0.0101^*^	0.0052	0.0017	0.0215	7.39%
		Conformity orientation → ASA W1 → ChIA W1 → CDI W1	0.0040^**^	0.0019	0.0013	0.0085	2.93%
Senior high school students	Conversation orientation	Total effect	−0.1589^***^	0.0204	−0.1990	−0.1188	
		Direct effect	−0.0213	0.0153	−0.0513	0.0087	13.40%
		Indirect effect	−0.1376^***^	0.0182	−0.1745	−0.1029	86.60%
		Conformity orientation → ASA W1 → CDI W1	−0.1202^***^	0.0173	−0.1556	−0.0879	75.65%
		Conformity orientation → ChIA W1 → CDI W1	−0.0141^*^	0.0067	−0.0292	−0.0030	8.87%
		Conformity orientation → ASA W1 → ChIA W1 → CDI W1	−0.0033	0.0025	−0.0083	0.0019	2.08%
	Conformity orientation	Total effect	0.1066^***^	0.0234	0.0606	0.1526	
		Direct effect	0.0071	0.0156	−0.0235	0.0377	6.66%
		Indirect effect	0.0995^***^	0.0195	0.0612	0.1393	93.34%
		Conformity orientation → ASA W1 → CDI W1	0.0914^***^	0.0181	0.0569	0.1285	85.74%
		Conformity orientation → ChIA W1 → CDI W1	0.0040	0.0060	−0.0075	0.0160	3.75%
		Conformity orientation → ASA W1 → ChIA W1 → CDI W1	0.0041^*^	0.0021	0.0005	0.0088	3.85%

***p < 0.001, **p < 0.01, *p < 0.05.

W1, baseline; W2, six-month follow up. Sex was coded as 0 for female and 1 for male in the correlation analysis.

ASA, the Anhedonia Scale for Adolescents; CDI, the Children’s Depression Inventory; ChIA, the Children’s Inventory of Anger.

Furthermore, for junior high school students, conformity orientation exhibited a direct positive effect on CDI scores (point estimate = 0.0676, *95% CI* = 0.0364 to 0.0988). In addition, the ASA total scores (point estimate = 0.0549, *95% CI* = 0.0266 to 0.0839) and ChIA total scores (point estimate = 0.0101, *95% CI* = 0.0015 to 0.0214) also independently mediated the relationship between conformity orientation scores and CDI score. The ASA and ChIA total scores sequentially mediated the association between conformity orientation scores and CDI scores (point estimate = 0.0040, *95% CI* = 0.0012 to 0.0085). For senior high school student, the indirect effect of ASA total scores (point estimate = 0.0914, *95% CI* = 0.0562 to 0.1282) and the sequentially indirect effect of ASA and ChIA total scores (point estimate = 0.0041, *95% CI* = 0.0004 to 0.0088) on CDI scores are significant. However, the direct effect of conformity orientation scores (point estimate = 0.0071, *95% CI* = −0.0235 to 0.0377) and the indirect effect of ChIA total scores (point estimate = 0.0040, *95% CI* = −0.0075 to 0.0169) on CDI scores were non-significant.

To validate the mediating effect observed in the cross-sectional data, we conducted a longitudinal sequential multiple mediation analysis. Conversation orientation and conformity orientation scores at W1 were entered as independent variables, ASA and ChIA total scores at W2 as mediators, and CDI scores at W2 as the dependent variable. As shown in Tables 3, 4, for both junior and senior high school students, conversation orientation at W1 had a significant indirect effect on depressive symptoms at W2. This significant indirect effect was attributable to ASA total scores, whereas the indirect effect of ChIA total scores and the sequential mediating pathway through ASA and ChIA predicting CDI scores were non-significant. Conformity orientation at W1 did not significantly predict ASA, ChIA, or CDI scores at W2 for both junior and senior high school students.

**Table 3:** Longitudinal model regression analysis of family communication patterns.

School stage	Family communication patterns	Dependent variable	Independent variable	*R* ^2^	*F*	*B*	*SE*	*t*	*p*
Junior high school students	Conversation orientation	Model 1		0.30	56.40				<0.001
		ASA W2	Conversation orientation			−0.09	0.03	3.05	<0.01
			Age			0.0.2	0.02	0.65	=0.51
			CDI W1			0.66	0.07	9.20	<0.001
		Model 2		0.13	14.74				<0.001
		ChIA W2	Conversation orientation			−0.05	0.03	−1.65	=0.10
			ASA W2			0.25	0.05	4.54	<0.001
			Age			−0.005	0.03	0.18	=0.85
			CDI W1			0.12	0.08	1.37	=0.17
		Model 3		0.71	192.57				<0.001
		CDI W2	Conversation orientation			0.005	0.01	1.18	=0.24
			ASA W2			0.45	0.02	19.45	<0.001
			ChIA W2			0.07	0.02	1.57	=0.12
			Age			−0.007	0.01	0.55	=0.58
			CDI W1			0.29	0.04	8.26	<0.001
	Conformity orientation	Model 1		0.29	52.45				<0.001
		ASA W2	Conformity orientation			−0.03	0.03	0.95	=0.34
			Age			0.0.2	0.03	0.73	=0.46
			CDI W1			0.75	0.07	11.44	<0.001
		Model 2		0.13	13.97				<0.001
		ChIA W2	Conformity orientation			−0.007	0.03	−0.21	=0.83
			ASA W2			0.26	0.05	4.84	<0.001
			Age			−0.006	0.03	0.19	=0.84
			CDI W1			0.18	0.08	2.18	<0.05
		Model3		0.71	191.70				<0.001
		CDI w2	Conformity orientation			0.005	0.01	0.37	=0.71
			ASA W2			0.45	0.02	19.39	<0.001
			ChIA W2			0.03	0.02	1.48	=0.14
			Age			−0.007	0.01	0.54	=0.59
			CDI W1			0.27	0.03	8.09	<0.001
Senior high school students	Conversation orientation	Model 1		0.37	65.83				<0.001
		ASA W2	Conversation orientation			−0.06	0.03	−2.29	<0.05
			Age			−0.11	0.03	−3.42	<0.001
			CDI W1			0.78	0.07	11.51	<0.001
		Model 2		0.08	7.22				<0.001
		ChIA W2	Conversation orientation			−0.05	0.03	−1.48	=0.14
			ASA W2			0.05	0.06	0.78	=0.44
			Age			−0.05	0.04	−1.35	= − 0.13
			CDI W1			0.26	0.09	2.83	<0.01
		Model 3		0.72	166.16				<0.001
		CDI W2	Conversation orientation			0.02	0.01	1.24	=0.21
			ASA W2			0.34	0.03	13.38	<0.001
			ChIA W2			0.09	0.02	4.27	<0.001
			Age			−0.01	0.02	−0.69	=0.49
			CDI W1			0.42	0.04	11.05	<0.001
	Conformity orientation	Model 1		0.37	63.84				<0.001
		ASA W2	Conversation orientation			0.03	0.03	1.21	=0.23
			Age			−0.11	0.03	−3.47	<0.001
			CDI W1			0.82	0.07	12.72	<0.001
		Model 2		0.07	6.69				<0.001
		ChIA W2	Conversation orientation			0.02	0.03	0.51	=0.61
			ASA W2			0.06	0.06	0.93	0.35
			Age			−0.05	0.04	−1.37	=0.17
			CDI W1			0.29	0.09	3.20	<0.01
		Model 3		0.71	165.48				<0.001
		CDI W2	Conversation orientation			0.01	0.01	0.75	=0.44
			ASA W2			0.34	0.03	13.23	<0.001
			ChIA W2			0.09	0.02	4.16	<0.001
			Age			−0.01	0.02	−0.66	=0.51
			CDI W1			0.48	0.04	10.82	<0.001

W1, baseline; W2, six-month follow up. Sex was coded as 0 for female and 1 for male in the correlation analysis.

ASA, the Anhedonia Scale for Adolescents; CDI, the Children’s Depression Inventory; ChIA, the Children’s Inventory of Anger.

**Table 4:** Longitudinal analysis of model mediating effect of conversation orientation.

School stage	Path	Effect	Boot SE	Boot LLCI	Boot ULCI
Junior high school students	Total effect	−0.0269	0.0185	−0.0632	0.0094
	Direct effect	0.0154	0.0130	−0.0103	0.0410
	Indirect effect	−0.0432^***^	0.0139	−0.0701	−0.0150
	Conversation orientation → ASA W2 → CDI W2	−0.0398^**^	0.0135	−0.0665	−0.0133
	Conversation orientation → ChIA W2 → CDI W2	−0.0017	0.0012	−0.0055	0.0010
	Conversation orientation → ASA W2 → ChIA W2 → CDI W2	−0.0007	0.0007	−0.0023	0.0003
Senioir high school students	Total effect	−0.0102	0.0162	−0.0420	0.0216
	Direct effect	0.0160	0.0129	−0.0094	0.0415
	Indirect effect	−0.0263^*^	0.0108	−0.0481	−0.0049
	Conversation orientation → ASA W2 → CDI W2	−0.0215^*^	0.0102	−0.0481	−0.0049
	Conversation orientation → ChIA W2 → CDI W2	−0.0045	0.0034	−0.0120	0.0016
	Conversation orientation → ASA W2 → ChIA W2 → CDI W2	−0.0003^*^	0.0006	−0.0017	0.0007

***p < 0.001, **p < 0.01, *p < 0.05.

W1, baseline; W2, six-month follow up. Sex was coded as 0 for female and 1 for male in the correlation analysis.

ASA, the Anhedonia Scale for Adolescents; CDI, the Children’s Depression Inventory; ChIA, the Children’s Inventory of Anger.

### 3.5 Sex difference in the relationship between family communication patterns and depression

To examine the role of sex in the aforementioned relationships, we conducted four moderated mediation analyses using SPSS PROCESS Macro (Model 92), assessing sex differences in the associations among family communication patterns, ASA total scores, ChIA total scores, and CDI scores in both cross-sectional and longitudinal data.

**Figure 1:**
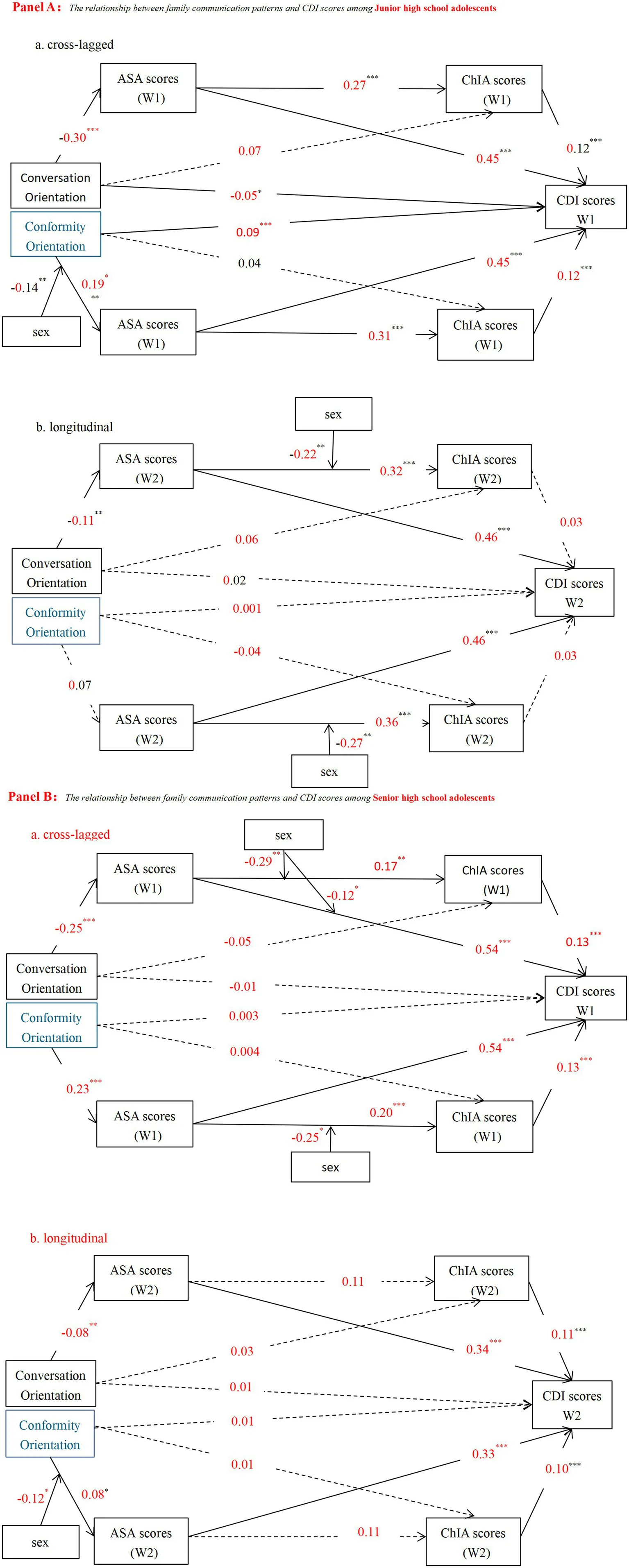
**Panel A**: The relationship between family communication patterns and CDI scores among Junior high school adolescents. **Panel B**: The relationship between family communication patterns and CDI scores among Senior high school adolescents. ***p < 0.001, **p < 0.01, *p < 0.05.

As shown in Figure 1, the results demonstrated that, based on longitudinal data, for junior high school students, the ASA total scores had a significant effect on the ChIA total scores among female participant, with point estimate = 0.3244, *95% CI* = 0.1913 to 0.4576. However, we did not find such a sex difference in the cross-sectional data of junior high school students. For senior high school students, the predictive path from ASA total scores to ChIA total scores was only statistically significant among male participants in cross-sectional data, with point estimate = −0.1615, *95% CI* = −0.2554 to −0.0676. In addition, the indirect effect of ASA in the relationship between conversation scores and CDI scores was stronger among female participants, with point estimate = − 0.1201, *95% CI* = −0.2276 to −0.0125. In longitudinal data, we we did not find such a sex difference.

As for conformity orientation among junior high school students, the results demonstrated that conformity orientation scores had a significant effect on ASA total scores for female participants in cross-sectional data, with point estimate = 0.1965, *95% CI* = 0.1187 to 0.2743. In addition, ASA total scores had a significant effect on ChIA total scores for female participants in longitudinal data, with point estimate = 0.3598, *95% CI* = 0.2300 to 0.4895. Among senior high school students, we found that ASA total scores had a significant effect on ChIA total scores for female participants in cross-sectional data, with point estimate = 0.2023, *95% CI* = 0.0865 to 0.3181. In addition, conformity orientation scores had a significant effect on ASA total scores for female participants in longitudinal data, with point estimate = 0.0797, *95% CI* = 0.0088 to 0.1507.

Regarding group differences between junior and senior high school students, distinct patterns emerged across cross-sectional and longitudinal data. In cross-sectional data, the results showed that family communication patterns had a small but significant direct effect on CDI scores among junior high school students, whereas this path did not significant for senior high school students. Significant group differences were also observed in the longitudinal associations between ASA total scores and ChIA total score, as well as between ChIA total scores and CDI scores. Among junior high school students, ASA total scores significantly predicted ChIA total scores, whereas ChIA total scores did not predict CDI scores. By contrast, among senior high school students, ASA total scores failed to significantly predict ChIA total scores, but ChIA total scores showed a significant predictive effect on CDI scores.

## 4 Discussion

This study employed a two-wave design to examine the relationships among family communication patterns, diminished hedonic capacity, anger expression, and depressive symptoms. Cross-sectional analyses revealed that diminished hedonic capacity and anger expression sequentially mediated the associations between both conversation and conformity orientations and depressive symptoms. Specifically, the sequential mediation was significant for conversation orientation among junior high school students and female senior high school students, and for conformity orientation among female junior and senior high school students. However, this mediation was not identified in the longitudinal analyses. Instead, the longitudinal analyses indicated that only diminished hedonic capacity mediated the relationship between family communication patterns and depressive symptoms for both junior and senior high school students.

Our findings align with previous research indicating that conversation orientation serves a protective role against adolescent depression (Tang et al., 2020; Keating, 2016; Curran and Allen, 2016; Huang et al., 2023). According to family communication patterns theory, conversation-oriented families encourage open expression, constructive conflict resolution, and emotional exchange between parents and adolescents (Koerner and Fitzpatrick, 1997; Koerner and Fitzpatrick, 2002). These positive interactions contribute to a cascade of psychological benefits, including reduced depressive symptoms (Huang et al., 2023; Zhou et al., 2023; Ioffe et al., 2020). In contrast, conformity-oriented families—marked by conflict avoidance, limited emotional support, and suppression of emotional communication—are associated with negative psychological outcomes, such as heightened depressive symptoms (Huang et al., 2023; Zhou et al., 2023). These results highlight the crucial role of family communication patterns in adolescent mental health. Shifts in communication style within the family can influence adolescents’ information processing, behavioral regulation, and emotional functioning (Schrodt et al., 2008). A supportive parent–child relationship fosters feelings of being understood and accepted, thereby reducing frustration and depressive symptoms.

We further found that diminished hedonic capacity and anger expression sequentially mediate the relationship between family communication patterns and adolescent depression. Specifically, this sequential mediating effect only existed for conversation orientation among junior high students and female senior high students. It is also worth noting that this sequential mediating effect only existed in cross-sectional data, and we only found that diminished hedonic capacity played a mediating role between family communication patterns and depression among junior and senior high school students. Cross-sectional data findings underscore the potential role of anger expression as a downstream consequence of diminished hedonic capacity, which in turn contributes to depressive symptoms. Prior research has shown that anhedonia is linked to impairments in physical, psychological, and social functioning in individuals with major depressive disorder (Wong et al., 2024), and that it is rooted in dysfunctions of the brain’s reward system (Der-Avakian and Markou, 2012). Drawing on the frameworks of the NAIT and the RDT, the observed sequential mediation becomes more theoretically grounded. As previously discussed, adolescents raised in conversation-oriented families are encouraged to express themselves freely (Koerner and Fitzpatrick, 2002), which enhances their ability to communicate goals and expectations effectively (Koerner and Fitzpatrick, 2002, 1997; Schrodt et al., 2008), and allows them to perceive external interactions more positively. According to NAIT and RDT, positive interpretation of external events is associated with the development of rewarding expectations (Barkus, 2021), a key component in enhancing hedonic capacity. In turn, increased hedonic capacity reduces anger expression, ultimately mitigating depressive symptoms. Conversely, adolescents from conformity-oriented families—where conflict avoidance and emotional suppression are emphasized—tend to exhibit poorer social skills (Schrodt et al., 2008), are more likely to perceive external experiences negatively, and show diminished hedonic capacity. This leads to heightened anger expression and, subsequently, more severe depression.

Regarding the results of longitudinal data analyses, we found that only diminished hedonic capacity exerted a mediating effect on the association between family communication patterns and depression across junior and senior high school students. These results revealed that family communication patterns exerted a stronger influence on adolescents’ diminished hedonic capacity relative to anger expression. The stable mediating role of diminished hedonic capacity among adolescents has been validated by Kuan et al. (2024). As mentioned before, family communication patterns shape the emotional climate within families and closely associated with various aspects of individual developments such as cognition, relationship anticipation, and communication apprehension (Koerner and Fitzpatrick, 2002). The typical characteristic of anhedonia is a lack of pleasurable experience and reduced responsiveness to rewarding stimuli (Serretti, 2023; Treadway and Zald, 2013), both of which are deeply influenced by family climate and prior experience. These findings are also consistent with the diathesis-stress model, suggesting that anhedonia-related reward-system-functioning deficits resulted in greater sensitivity to family communication patterns, which in turn accounts for their association with depressive symptoms (Borsini et al., 2020). A large body of evidence demonstrates that anhedonia is associated with reduced ventral striatal activation in response to reward receipt (Pizzagalli, 2022). These results indicated that we should pay more attention to adolescent’s diminished hedonic capacity, which exerts a relatively strong effect on their depressive symptoms.

Taking school stage and sex into consideration, the relationship among family communication patterns, anhedonia, anger expression, and depression became more complex. Regarding sex differences, the results revealed that conformity orientation exerted stronger adverse effects on girls than on boys among both junior and senior high school students. These findings align with previous research suggesting that girls are more sensitive to the quality of their family environment (Zhou et al., 2023). Compared to boys, girls tend to place greater emphasis on maintaining harmonious familial relationships and invest more emotionally in these dynamics. As a result, they may be particularly vulnerable to psychological difficulties when family communication is ineffective (Crawford et al., 2003; Davies and Windle, 1997), which can manifest as heightened anhedonia, increased anger expression, and, ultimately, elevated depressive symptoms. We also found that the relationship between anhedonia and anger expression was stronger in girls than in boys. This is consistent with prior studies reporting a higher frequency of anger expression among girls (Judd et al., 2013; Fischer and Roseman, 2007). According to social appraisal theory, girls are more likely to express anger when they perceive it as justified (Fischer and Evers, 2011; Evers et al., 2011), and diminished hedonic capacity may serve as such a trigger—especially within emotionally unsupported environments. Girls may also be more attuned to the absence of emotional anticipation in social contexts, potentially using anger as a coping strategy for diminished reward experiences (Zhang et al., 2020). In supportive family environments, girls may be protected from depression through enhanced hedonic capacity and reduced anger expression. Conversely, in conformity-oriented or dysfunctional families, they may struggle to experience positive emotions, leading to diminished hedonic capacity, greater anger expression, and increased risk of depression. These findings underscore the critical importance of promoting positive family communication, particularly for adolescent girls.

Regarding school stage differences, we found the sequential mediating effect of diminished hedonic capacity and anger expression between conversation orientation and depression was significant for junior high school students, while the sequential indirect effect was only statistically significant for female senior high students. These results can be interpreted from gender differences in emotion expression with increasing age (Chaplin and Aldao, 2013). Chaplin and Aldao (2013) found that boys engaged in less externalizing emotional expression relative to girls. Moreover, male adolescents are educated to suppress emotions (Ahmmed and Khan, 2024). It is reasonable to infer that male senior high school students learn to suppress their emotions, which breaks the mediating pathway linking diminished hedonic capacity to anger expression. These results indicated that interventions targeting adolescent depression should take school stage and sex into consideration.

### 4.1 Limitations and future direction

This study has several limitations that should be acknowledged. First, the sample consisted exclusively of non-clinical adolescents, which limits the generalizability of the findings to clinically diagnosed populations. Future research should examine how family communication patterns influence the onset and progression of depression in clinical samples. Second, family communication patterns were assessed only at baseline and treated as stable traits, which constrains interpretation and overlooks the potential bidirectional relationship between family dynamics and adolescent depression. Longitudinal studies with repeated assessments of family communication are needed to explore these reciprocal influences. Third, the study relied solely on self-reported measures, without incorporating objective or physiological indicators. Prior research has linked anhedonia and depression-related behaviors to diminished activity and reactivity within dopaminergic neural systems (Treadway and Zald, 2013). Future studies should investigate whether family communication patterns affect adolescent depression through underlying neurobiological mechanisms, particularly those related to hedonic capacity and anger regulation.

## 5 Conclusion

In conclusion, this study offers compelling evidence that adolescents from conversation-oriented families are less likely to exhibit elevated depressive symptoms, with diminished hedonic capacity and anger expression serving as key mediating mechanisms. Notably, diminished hedonic capacity exerts a far more stable mediating effect than anger expression. In addition, for senior high school students, this sequential indirect effect was only significant among female adolescents. In contrast, conformity orientation is associated with higher levels of depressive symptoms, particularly among girls. This study found that the sequential mediating pathway—diminished hedonic capacity followed by increased anger expression—was significant only for female participants in the context of conformity-oriented communication. These findings underscore the importance of fostering conversation-oriented family communication as a potential target for interventions aimed at mitigating adolescent depression, with particular attention to the needs of girls.

## Data Availability

The raw data supporting the conclusions of this article will be made available by the authors, without undue reservation.
